# Bridging the gap between microbial limits and extremes in space: space microbial biotechnology in the next 15 years

**DOI:** 10.1111/1751-7915.13927

**Published:** 2021-09-17

**Authors:** Charles S. Cockell

**Affiliations:** ^1^ UK Centre for Astrobiology School of Physics and Astronomy University of Edinburgh Edinburgh UK

## Abstract

The establishment of a permanent human settlement in space is one of humanity’s ambitions. To achieve this, microorganisms will be used to carry out many functions such as recycling, food and pharmaceutical production, mining and other processes. However, the physical and chemical extremes in all locations beyond Earth exceed known growth limits of microbial life. Making microbes more tolerant of a greater range of extraterrestrial extremes will not produce organisms that can grow in unmodified extraterrestrial environments since in many of them not even liquid water can exist. However, by narrowing the gap, the engineering demands on bioindustrial processes can be reduced and greater robustness can be incorporated into the biological component. I identify and describe these required microbial biotechnological modifications and speculate on long‐term possibilities such as microbial biotechnology on Saturn’s moon Titan to support a human presence in the outer Solar System and bioprocessing of asteroids. A challenge for space microbial biotechnology in the coming decades is to narrow the microbial gap by systemically identifying the genes required to do this and incorporating them into microbial systems that can be used to carry out bioindustrial processes of interest.

## Abstract 2

A challenge for space microbial biotechnology in the coming decades is to narrow the microbial gap between the growth limits of known life and conditions to be found in extraterrestrial environments.

## Introduction

The next 15 years will see an increasing effort in the exploration of space, both robotic and human. This will be partly driven by the improvement of rocket technology to explore space, once a feat of wizardry only accomplishable by the large nation states of the Cold War, and the entry of the private sector into one of the most rate‐limiting problems in space exploration – access to Earth orbit and beyond (Musk, 2017).

In particular, with respect to human exploration efforts, there is an increasing interest in using microorganisms to perform many functions that will be required to permit humans to live temporarily in space, such as in Earth orbit, or to build more permanent stations in locations such as the Moon or Mars (Horneck, 2000; Cockell, 2010; Horneck *et al*., 2010; Montague *et al*., 2012; Menezes *et al*., 2015; Moissl‐Eichinger *et al*., 2016; Rothschild, 2016; Berliner *et al*., 2021). For instance, given the paucity of water and carbon atoms in many places, such as the Moon, recycling is essential, and microbial isolates and communities can be used to degrade and recycle waste to recover important elements (Menezes *et al*., 2015), such as phosphorus and carbon. Microorganisms will be used as food, for example in cyanobacterial and algal biomass, and to produce ubiquitously required oxygen gas through photosynthesis (Billi *et al*., 2013). Engineered microorganisms can be used to produce a wide range of high value products such as pharmaceuticals and plastics and they might even be used in construction (Montague *et al*., 2012; Berliner *et al*., 2021) or in biomining of economically useful elements (Cockell *et al*., 2020; Volger *et al*., 2020).

In many ways, we might simply observe that the multiplicity of functions and services provided by microorganisms on Earth, both in natural ecosystems and in artificial bioindustrial processes, could be replicated in space to supply human needs. An additional spur to this high demand for microbial processes in space is that microorganisms can minimize the volume, mass, and energy associated with many of the aforementioned processes and microbial reproduction can reduce the quantity of material that must be shipped from Earth to supply local requirements, allowing a station to eventually become self‐sustaining. Furthermore, microorganisms, by using local resources, for example carbon dioxide in the Martian atmosphere to produce oxygen, would aid in achieving the capacity to ‘live off the land’, known as In‐Situ Resource Utilization (ISRU). This too enhances self‐sufficiency.

**Fig. 1:**
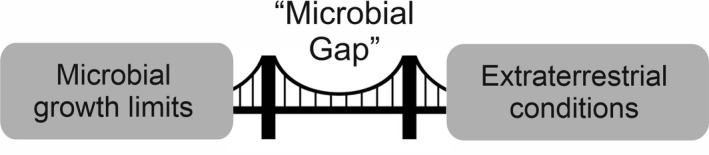
The Microbial Gap. There is a gap between the limits of growth of known microorganisms and extraterrestrial conditions. By modifying microorganisms to be more closely matched to the extraterrestrial conditions of interest, this gap can be partially (though usually not wholly) bridged. By reducing the gap using biotechnology, the subsequent engineering challenges to close the gap can be relaxed and greater robustness introduced into bioprocessing capabilities beyond Earth.

However, there is a challenge that lies at the core of all of these microbial processes, depicted in Fig. 1. The extreme conditions to be found in space mean that no known microorganism can live in natural extraterrestrial settings where humans might operate. For example, on the Moon, the lack of an appreciable atmosphere means that liquid water cannot persist on its surface, thus rendering it uninhabitable to all known life, which requires liquid water as a biochemical solvent (Pohorille and Pratt, 2012). This liquid water deprivation is also true for other locations, such as interplanetary space and the surface of Mars. In the latter case, the surface sits precariously on the triple point (the pressure and temperature conditions at which all three phases of matter can co‐exist), making it equivocal that liquid water might persist in certain places temporarily for certain times of the year (Martin‐Torres *et al*., 2015). Other challenges in many locations in space include high ionizing radiation (Nelson, 2016) and on the surface of Mars, potentially toxic ions such as perchlorate salts (Hecht *et al*., 2009).

We can observe, therefore, that there exists a ‘microbial gap’ between the growth limits of known microbial life (Merino *et al*., 2019), and the conditions under which we would like to operate in any extraterrestrial environment. There are candidate locations in the Solar System that might potentially be habitable, such as the subsurface of Mars (Michalski *et al*., 2013) and the liquid interior of icy moons (e.g. McKay *et al*., 2008) in which the microbial gap would be closed but humans are not expecting to establish stations in those places.

One objective of microbial biotechnology in the next fifteen years and further beyond will be to engineer an increasing number of microorganisms that narrow the microbial gap. This objective would produce a number of advantages, three of which are: (i) it would reduce the engineering demands required to close the gap. For example, photosynthetic microorganisms that can grow under high radiation and lower than one atmosphere pressure on Mars would require a greenhouse structure that is less radiation attenuating (potentially less thick) and in which the pressure differential with the outside environment would be less extreme than that required for operating at one atmosphere (0.1 MPa), thus reducing the engineering challenge for the design of the bulk structure; (ii) microorganisms optimally engineered or synthetically fabricated to grow under more extreme conditions can potentially be more efficient in their use of resources, such as nutrients, and have greater yields when grown closer to extraterrestrial conditions; and (iii) by creating organisms with high resistance to diverse extraterrestrial extremes, either in dormant or growth stages, the microbial components of any extraterrestrial station can be more robust to exposure to such extremes. For example, they could be made less likely to lose viability, or suffer damage, either during growth or in storage as a result of unplanned failures in structures enclosing them. This may be critical where humans are dependent on these microbial processes, such as in waste recycling or oxygen production.

In this paper, I treat the microbial gap as different from the subsequent modifications (such as the introduction of genes) needed to carry out desired industrial processes such as plastics or pharmaceutical production. Here, I am concerned with the core adaptations required to bring microbial physiological capacities closer to environmental conditions, not the additional biotechnological modifications that are needed to carry out the chosen range of industrial processes that might be useful in space. This approach bears some similarities to the interest in enhancing the physiological fitness of microbial cell lines used in terrestrial biotechnological processes operating under physical and chemical extremes (Zhang *et al*., 2009; Gong *et al*., 2017). I also emphasize that in this paper I focus on the modifications we desire, giving some potential examples, and not on the means by which these changes are to be achieved, which could be by genetic modification techniques, synthetic biology approaches or adaptive laboratory evolution (ALE). The approach used will clearly depend on the modifications required and their extent. Furthermore, in the future it may be possible to arrange extraterrestrial systems whereby organisms exposed to these conditions self‐evolve, being selected in situ by robotic means for their improved performance. In this case, prior modification to withstand extraterrestrial conditions would then be augmented with further fine‐tuning in the environment of interest. Finally, many industrial processes involve consortia of microorganisms, such as waste recycling and biomining, implying that the modifications I suggest could be required in a range of organisms applied to a given process.

In this perspectives piece, I want to discuss some of the challenges and suggest some ways to create a programme of work to address the problem of the microbial gap in space microbial biotechnology.

## Common challenges with the microbial gap

There are many potential environments in which humans already have established, or plan to establish, temporary or permanent settlement in our Solar System. They include Earth orbit, orbits around the Moon, the lunar surface itself, Martian orbits and the Martian surface. In the much longer term, we might consider the surface of Jovian and Saturnian moons, and destinations beyond these bodies, including the Oort cloud.

**Fig. 2:**
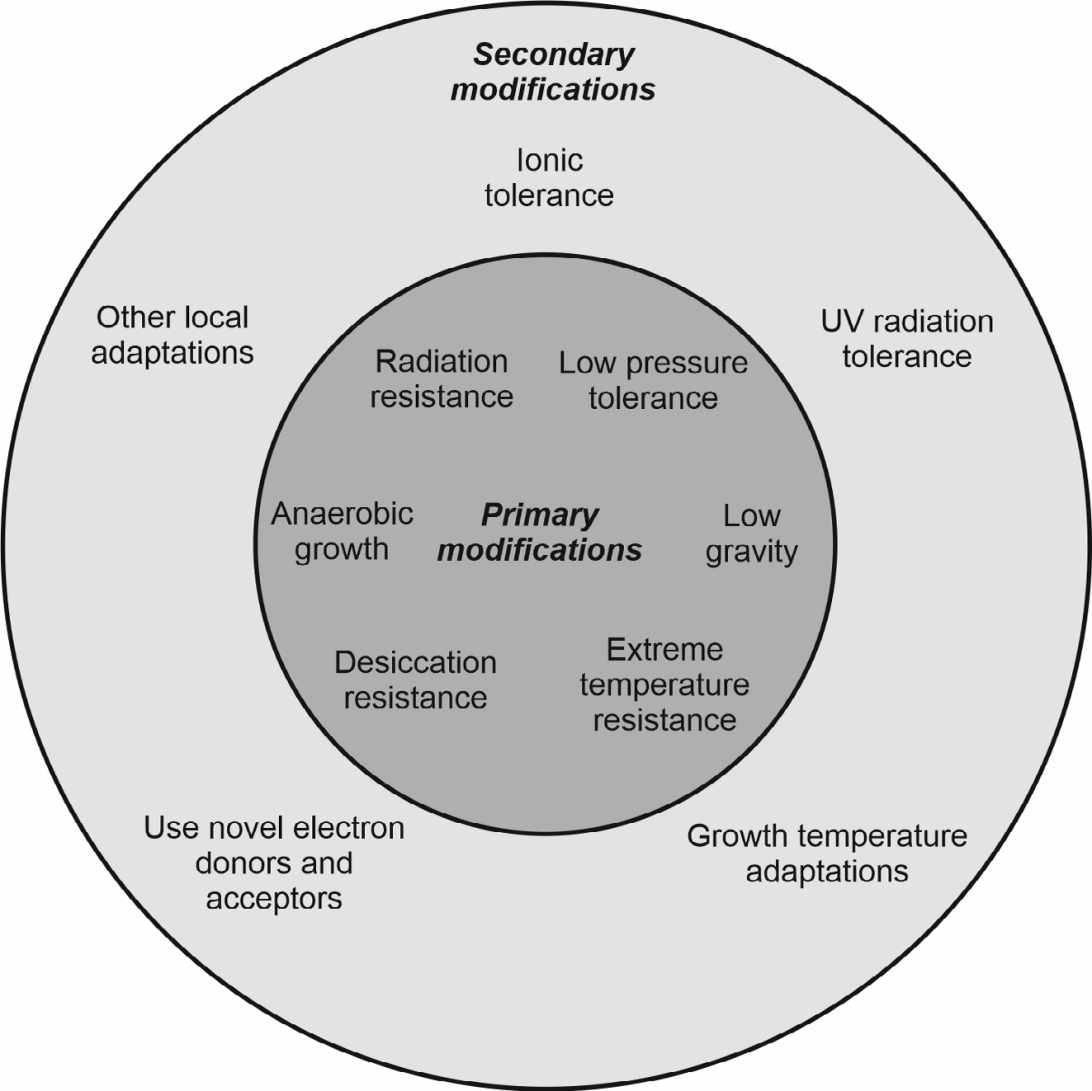
Biotechnological adaptations of microorganisms for space settlement. There are some adaptations that are applicable to most environments in space (‘primary’ modifications). A range of adaptations could be introduced that improve microbial adaptations to selected stressors associated with specific environments (‘secondary’ modifications). Some examples are provided here.

Yet despite these remarkable and apparently endless possibilities, from a microbial perspective they all impose some common physiological challenges and so we could say that fortuitously for humanity, a limited, selected set of capabilities engineered into microorganisms will go some way to bridging the microbial gap for a wide range of future exploration scenarios. Six of those key challenges are radiation resistance, low pressure tolerance, periodic desiccation tolerance, growth temperature extremes, anaerobic growth and low gravity. We might think of these as the ‘primary’ microbial adaptations we would be interested in because of their ubiquity (Fig. 2). Some observations on each of these are merited.

### Radiation resistance

In most locations beyond Earth where humans might eventually establish a presence, radiation levels are higher than on Earth. For example, on the International Space Station, annual radiation exposure is ~ 114 mSv, compared with Earth, which is 6.2 mSv (Cucinotta *et al*., 2008). On the Moon, radiation levels of ~ 500 mSv per year have been measured (Zhang *et al*., 2020). Levels on Mars are ~ 230 mSv per year (Hassler *et al*., 2014). The daily dose on Jupiter’s moon Europa is several Sv per day because of the intense radiation within Jupiter’s magnetic field (Baumstark‐Khan and Facius, 2002). Organisms in space, unprotected by a thick atmosphere, can also be exposed to Solar Particle Events, which are ejections of protons from the Sun that can increase radiation levels by several fold (Baumstark‐Khan and Facius, 2002).

We do know that some microorganisms possess extreme radiation tolerance. For example, the thermophile *Thermococcus gammatolerans* can resist up to 30 kGy of γ‐radiation (Jolivet *et al*., 2003) and similar radiation‐resistant capacities are reported for the mesophilic *Deinococcus radiodurans* (Battista *et al*., 1999) and the cyanobacterium *Chroococcidiopsis* sp. (Billi *et al*., 2000a). *D*. *radiodurans* can survive chronic radiation exposure of ~50 Gy per hour (Daly *et al*., 2004), in excess of typical extraterrestrial exposures. The biochemical basis of radiation resistance is not fully understood, but it seems that it cannot be localized to one specific capacity but involves a range of responses including upregulation of pigments and proteins capable of quenching reactive oxygen species (ROS) and free radical products of Fenton reactions, enhanced DNA repair capabilities, multiple genome copies, and the accumulation of manganese (Daly *et al*., 2004; Daly, 2009; Krisko and Radman, 2010).

The ubiquity of higher radiation levels beyond Earth would suggest that microbial biotechnological work to enhance or augment radiation tolerance in a wide diversity of microorganisms required in space is a priority. First, it is necessary to identify the genetic basis of the multiple responses that radiation‐resistant microorganisms have and then to determine the extent to which these capacities can be generically inserted into new organisms to confer such resistance, in other words how much taxa‐specific engineering is required to confer radiation resistance. Clearly, for efficiency, the optimum outcome of such biotechnological effort would be a core cassette of genes that could be incorporated into as many microbial taxa as possible with as little specific modification as possible. Alternatively, another approach is to use radiation‐resistant taxa, such as *Deinococcus*, as a chassis for incorporating industrial processes of interest, an approach whose efficacy has been demonstrated (Gerber *et al*., 2015). Directed evolution approaches could be used with some organisms to enhance resistance.

### Low pressure tolerance

On the Moon, the surface is exposed to a vacuum, similarly to all other small, essentially atmosphere‐free, bodies such as asteroids. On Mars, the average surface pressure is 0.6 kPa, at the triple point. Although we cannot engineer microorganisms to grow exposed in these environments, since water will freeze and sublimate, it would be of benefit to be able to enhance their capacity to grow optimally under low pressures, thus reducing the pressurization required in fermentation reactors, and any microbial growth apparatus, which is situated in the ambient environment. This will reduce the structural problems of extreme pressure differentials between the inside and outside of the facility. Similar motivations to reduce the pressure differential in planetary organismal growth facilities have been suggested for plants and insects. Work on plants suggests that greenhouses could be run at ~20‐30 kPa (Boston, 1981; Andre and Massimino, 1992). In the case of insects, they were shown to be able to function at ~ 1 kPa under Earth atmospheric composition, although under stress (Cockell *et al*., 1999). The optimum operating pressure of any facility will depend on the organisms enclosed and the complexity of the community.

Low pressure is not a recognized microbial stress on Earth; high pressure is a better understood stress (Bartlett, 2002). However, it is clearly the case that microorganisms grown under low pressure experience stress (Nicholson *et al*., 2010; Fajardo‐Cavazos *et al*., 2018). The extent to which low pressure effects operate directly on microorganisms or indirectly, for instance by changing solution gas chemistry, is still not fully understood. Differential effects on microbial growth at low partial pressures of oxygen compared with low total pressure show that low pressure exerts distinct effects, separate from merely hypoxia. Changes in membrane composition, such a decrease in the unsaturated to saturated lipid ratio at low pressures may reflect a low pressure increase in membrane fluidity and permeability, with cells attempting to accommodate or reduce these low pressure‐induced effects (Fajardo‐Cavazos *et al*., 2012).

From the point of view of microbial biotechnology, one might try to identify the range of direct and indirect effects caused by a low‐pressure regime and then identify the genetic adaptations required to enhance microbial growth under those given stresses. With such knowledge, ‘low pressure tolerance’ might be engineered into microorganisms. In this way, the structural costs and complications of extreme pressure differentials in bioreactors and other facilities housing microbial processes can be reduced as well as potentially the resources in gases that would otherwise be required to maintain a 0.1 MPa pressure inside growth units.

Like radiation tolerance, low pressure tolerance is not an absolute requirement for humans to succeed beyond Earth. In a similar way in which radiation can be mitigated by burying a station in thick enough soil and rock, low pressures can be avoided by accepting the engineering costs of ubiquitous one atmosphere pressure differentials with the external environment. However, consistent with Fig. 1, by closing the gap using biotechnological approaches, these engineering requirements can be relaxed.

Yet another application of low pressure tolerance is in the use of local atmospheric resources on Mars. For example, the partial pressure of nitrogen on Mars has been shown experimentally to be too low for biological nitrogen fixation in *Azotobacter* and *Azomonas* spp. (Klingler *et al*., 1989). Nitrogen constitutes 2.7% of the atmosphere that is already at the low total pressure of 0.6 kPa. Yet if we wish to use nitrogen fixation to produce fixed forms of nitrogen for other microorganisms and plants in biotechnological applications (Berliner *et al*., 2021), which are lacking in Martian regolith (the rocks and material that make up the surface), then we need to be able to fix it. One approach would be to pressurize the Martian atmosphere and/or increase the nitrogen concentration by physical‐chemical means to achieve partial pressures conducive to biological nitrogen fixation in the desired microbial candidates. Another approach is to use biotechnology to narrow the microbial gap of diazotrophy by producing microbial systems capable of sequestering and fixing nitrogen gas at ambient Martian partial pressures or at nitrogen partial pressures generated by pressurizing Martian atmosphere to a minimum without further compositional modification.

### Periodic desiccation tolerance

There are scenarios in which microorganisms could be exposed to extreme low pressure or vacuum conditions, either deliberately in storage or accidentally in a depressurization event. This would be potentially deleterious if this was to occur with organisms upon which a life support system depended. Given that planetary stations are at all times surrounded by the conditions that make microbial desiccation instantaneously possible, it would seem prudent to engineer these capacities into any organisms destined for use in space. This way, greater resilience can be incorporated into biological components of a human outpost.

One characterized desiccation tolerance mechanism is the production of sugars such as trehalose, which protects biomolecules, for example from ROS, and stabilizes membranes (Leslie *et al*., 1995; Reina‐Bueno *et al*., 2012; Tapia *et al*., 2015) among other effects. Organisms may not have time to upregulate such molecules if they are exposed to these extremes rapidly (for example, on the order of minutes in a depressurization event). However, they could be engineered to produce these compounds constitutively to give them some inherent resistance against rapid desiccation. The introduction of the cyanobacterial sucrose‐6‐phosphate synthase (*SpsA*) gene into *Escherichia coli* has been shown to enhance tolerance of desiccation, including by freeze‐drying, by 10,000‐fold demonstrating the potential for biotechnological modification of organisms to confer desiccation resistance (Billi *et al*., 2000b). Similar results were also shown for *Pseudomonas putida* (de Castro *et al*., 2000). Furthermore, the production of these compounds could be engineered into cells to allow them to be dried down under extraterrestrial desiccation for storage purposes with minimal loss of viability.

### Extreme temperature tolerance

As with desiccation, accidental exposure of organisms to ambient temperatures would potentially expose them to extreme low (freezing) and high temperatures. The mean temperature on Mars is about –60°C and although equatorial temperatures can exceed 20°C, in general Mars is a low temperature environment. On the Moon, low temperatures reach about −170°C, similarly in interplanetary space. Although not a problem for Mars, a large number of space locations, when exposed directly to solar radiation, also experience extreme high temperatures. For example on the Moon, temperatures can exceed 125°C. These extremes are outside known microbial growth limits (Takai *et al*., 2008). Nevertheless, the engineering of organisms to tolerate temporary exposure to extreme low and high temperatures, such as in storage, would enhance the robustness of stored cultures, reducing their frailty to accidental exposure to ambient or near‐ambient conditions for short periods of time.

As with desiccation tolerance, freezing tolerance can be conferred by the production of sugars such as trehalose (Duong *et al*., 2020). Intriguingly, trehalose was also observed to confer resistance to high temperature exposure, presumably by protecting biomolecules from denaturation (Vargas *et al*., 2008; Reina‐Bueno *et al*., 2012). Trehalose production is likely not the only biotechnological route that could be developed, but it provides an example of an intervention that can yield cells resistance to multiple factors of relevance to the space environment.

An ideal biotechnological approach would be to engineer organisms with the minimum number of alterations required to achieve tolerance to desiccation, freezing and/or high temperatures, thus achieving general resistance to a suite of extraterrestrial ambient conditions. Approaches to generating multiple resistances are discussed by Gong *et al*. (2017) and might include attempts to manipulate global transcription controls or generic changes to membrane composition that confer improved resistance to multiple extremes.

### Anaerobic growth

Anaerobic growth requirements are not a particular problem in space as such, in that many biotechnological processes on Earth are accomplished in fermentative bioreactors and considerable focus is already given to their improvement (Demain, 2000; Parekh *et al*., 2000). The problem in space is more the challenge in carrying out any aerobic biotechnological processes because of the difficulty of acquiring oxygen molecules. They can be acquired by electrolysis of water, but water itself, such as on the Moon, can be a rare commodity. Any process that can avoid the use of oxygen will achieve considerable logistical, energy, and process cost savings.

I include anaerobic growth in primary modifications to emphasize that optimally we require *all* organisms being used in space operations to be able to use minimal quantities of oxygen or to do without the gas entirely. In cases where the process is already anaerobic then clearly no technological modifications are required; however, a space microbial biotechnology programme might focus on modification of aerobic or microaerobic organisms to be capable of carrying out anaerobic transformations (e.g. Schmitz *et al*., 2015; Kampers *et al*., 2021) or alternatively, industrially useful biochemical transformations currently found in aerobic strains should be modified and incorporated into an anaerobic chassis.

### Low gravity

All environments beyond Earth where we might want to establish a human presence have lower gravity than Earth. On the International Space Station microgravity prevails, which is essentially, for all practical purposes, a near absence of gravity. The gravity on the Moon is one‐sixth and on Mars three‐eighths that on Earth. Asteroids have gravity regimes at most a third that on Earth (such as on Ceres), but generally much lower depending on their size.

Altered gravity conditions, such as microgravity, have been shown to influence microbial growth and metabolic processes (e.g. Huang *et al*., 2018). The ability of prokaryotes to directly sense gravity remains a point of discussion; however, gravity can influence sedimentation and convection in bulk fluids, thus changing the local conditions for microbial growth. By allowing for thermal convection and sedimentation, gravity is thought to affect the mixing of nutrients and waste, thereby influencing microbial growth and metabolism. For example, the lack of fluid mixing in microgravity may cause waste build up around cells and a lack of new nutrient availability, potentially stressing cells. This problem could be obviated by mixing in bioreactors or alternatively, microbial motility might overcome some of the limitations of static water in microgravity. The reported effects on microbial growth are contradictory with both improvements and reductions in growth rates reported in different organisms (Gasset *et al*., 1994; Kacena *et al*., 1999; Leys *et al*., 2004; Crabbé *et al*., 2013; Foster *et al*., 2014; Santomartino *et al*., 2020).

Our understanding of partial gravity is also poor. In a recent experiment in a biomining experiment onboard the International Space Station, final microbial cell numbers were found to be similar in microgravity, and simulated Martian and Earth gravity (Santomartino *et al*., 2020). These data were explained by the relatively long‐term duration of the experiment (21 days) with respect to the microbial growth rates leading to similar cell numbers in stationary phase, regardless of gravity. Fractional gravity levels have been shown to have differential effects on organisms such as flagellates (Häder *et al*., 1996).

The varied results of micro‐ and partial gravity experiments make it difficult to suggest a coherent biotechnological programme, in other words to identify a set of genes that would improve microbial growth and product yield in given gravity conditions. Ultimately, they may depend on the strain and the process being investigated. Certain problems, such as static fluid regimes on low gravity objects like asteroids, might be overcome by introducing motility genes, engineering microorganisms to be able to cope with low nutrient fluxes into the cell, or to cope with localized waste build up around a cell without detrimentally affecting yields of bioproduction. These might involve changes in the cell envelope or proteins involved in the cell interaction with the extracellular medium as suggested for *D*. *radiodurans* (Ott *et al*., 2019).

Low gravity as a primary modification is raised here to draw attention to the fact that gravity is a physical factor beyond Earth that is ubiquitously different from the surface of the Earth. Its effects on fluid behaviour, and hence microbial biotechnological processes, merit a focus on it as another parameter that needs to be investigated for any given process, and genetic modifications implemented where they might allow for improvements in yield.

## Closing the gap for specific taxa

There are physico‐chemical stresses on other planetary bodies that are not outside the limits of known life as such, but will exceed the limits of specific organisms. Thus, although the gap as depicted in Fig. 1 has been bridged by evolution, we might want to artificially narrow that gap for selected microorganisms of industrial use that would not ordinarily possess those capacities. I consider four examples of this class of adaptations here.

### Ionic tolerance

One promising direction in space settlement is the use of local rocks and other surface material as a source of useful elements and minerals in a range of processes that include biomining, the provision of nutrients for life support systems, and as growth substrates for plants and their associated microbiome (Montague *et al*., 2012). These materials can contain ions that present challenges for microorganisms. For example, on Mars, in some locations the regolith will contain sodium chloride (Osterloo *et al*., 2010), various sulphate salts (Bibring *et al*., 2006), perchlorates (Hecht *et al*., 2009), and potentially combinations of these. Although none of these constituents is yet known to be present at concentrations that exceed known limits to life, it is possible that in localized regions certain ions could impose absolute limits to life, just as they do on Earth, for example high concentrations of magnesium and chloride ions (Hallsworth *et al*., 2007).

Despite the possibility of local conditions that render regolith uninhabitable, in most cases ions are likely to be sub‐lethal and we could imagine a biotechnological effort to engineer useful microorganisms to tolerate or even use these ions in their metabolic processes. An example would be to introduce halophily into organisms of interest to allow them to grow in regolith from regions on Mars with a high sodium chloride concentration. This might be achieved by engineering salt‐in adaptations in organisms, or altering them to be capable of producing a range of compatible solutes that would enhance salt tolerance (Shivanand and Mugeraya, 2011). Another example of a biotechnological modification to use ions would be engineering organisms in anaerobic processes to use perchlorate ions as a terminal electron acceptor to conserve energy for growth (Davila *et al*., 2013), transforming a potential Martian toxin into part of a redox couple.

Beyond Mars, we might consider the icy moons of the giant gas planets that likely have ices mixed with salts, including sulphates and chloride ions (Vu *et al*., 2016; Zolotov, 2017). They include the Jovian moon Europa, the Saturnian moon Enceladus, and even asteroids such as Ceres. Here too, microorganisms will not grow under ambient conditions, but if icy material from the crusts of these moons was melted and used as a fluid for industrial processes, then engineering microorganisms to grow in the presence of any salt present, thus obviating the need to filter the liquid of its natural ionic burden, would clearly be advantageous. A specific example might be the use of melted material from the surface of the Jovian moon Callisto, which has been suggested as a location for a human station on account of its ancient stable surface and lower radiation flux compared with other Jovian moons (Troutman *et al*., 2003). These possibilities illustrate the need to enhance collaboration between planetary science and microbial biotechnology. In this case, we need more information on the ionic composition of the Callisto surface material to be able to direct a microbial biotechnology programme towards the engineering of microorganisms to be able to grow in the presence of this liquefied material. Concomitantly, microbial biotechnologists can provide mission designers with the important physical and chemical parameters that need to be measured.

It is noticeable that the range of these ‘contaminant’ ions is quite limited. The sulphate and chloride anions are common throughout the Solar System. Perchlorate ions are mainly localized to Mars. Common cations at high concentrations are those associated with silicate rocks such as magnesium, potassium, calcium, sodium and iron. Thus here too, a limited repertoire of ions and tolerance to them could be a focus of biotechnological remedy, which would open up a large range of extraterrestrial potentialities.

### Ultraviolet (UV) radiation tolerance

Protected by an ozone shield, the surface of the Earth in general experiences lower levels of biologically damaging ultraviolet (UV) radiation compared with extraterrestrial environments exposed to solar radiation. For example, the surface of Mars experiences a DNA biologically effective irradiance about three orders of magnitude greater than Earth (Cockell *et al*., 2000), explaining the predicted rapid loss of viability of organisms on the surface of that planet (Schuerger *et al*., 2006). Without an atmosphere, similar observations apply to the Moon where the UV flux is more intense and to objects in the asteroid belt, which although further away than Mars, experience the unattenuated solar flux on Sun‐facing sides that result in biologically effective irradiances higher than Earth.

Unlike ionizing radiation, UV radiation is not deeply penetrating, being easily attenuated by microns of soil (Mancinelli and Klovstad, 2000). However, there may be reasons to require exposure of organisms to solar radiation, the most prominent being photosynthetic processes using natural solar radiation. Although artificial light can be used to drive this process, natural solar radiation can freely be acquired. The UV radiation component of solar radiation can be relatively easily preferentially removed (window glass removes short‐wavelength UV radiation). However, biotechnology might be used to enhance UV radiation resistance of microorganisms to reduce the stringency of UV screening required, for example by improving the efficacy of UV repair mechanisms such as excision repair and photolyase lesion removal (Goosen and Moolenaar, 2008).

A more exotic possibility is to engineer microorganisms to make use of UV radiation in energy acquisition. For example, organisms might be engineering to fluorescence absorbed UV radiation into visible wavelengths to drive photosynthesis. Although this effect is reported for long‐wavelength UVA (320–400 nm) radiation (Mantha *et al*., 2001), the search for, or engineering of, existing pigments capable of absorbing short‐wavelength UV radiation in the UVC (200–280 nm) and UVB regions (280–320 nm) relevant to extraterrestrial environments so as to emit visible wavelengths would be a potential way to transform damaging UV radiation into useful wavelengths of light that can power photosynthesis.

### Growth temperature adaptations

The optimum growth temperature of any microbial process is nominally set by the process itself. For example, degradation of waste may be accomplished by thermophiles growing at the high temperatures at which organic degradation may be favoured (Farges *et al*., 2008).

The engineering of low and high temperature growth capacity cannot exceed the limits for life. Currently, the lower limit to life is not precisely established, but reproduction is thought to be limited below about –15°C (Junge *et al*., 2004). The upper temperature limit is currently 122°C (Takai *et al*., 2008). These limits may change in the future, but they are unlikely to exceed the temperature extremes associated with many extraterrestrial environments discussed earlier.

However, as with the pressure differential challenge, the closer the optimum temperature of microbial processes can be made to ambient temperatures, the less energy and thermal regulation will be required. Furthermore, a more cosmopolitan temperature range for any organism will allow for less exacting temperature control in the process of interest and it can be used to reduce the chances of loss of viability in the event of unplanned temperature excursions or temporary loss of thermal control. On Earth, the temperature engineering of microorganisms has generally not been a priority. Instead, microorganisms with existing optimum temperature tolerances are engineered to carry out desired functions (e.g. Zeldes *et al*., 2015). However, in space, a critical dependence of human survival on certain life support processes may make engineering broad temperature ranges for growth, thus allowing engineering requirements to be less exacting, and system failures less likely to result in loss of organisms, a useful objective of biotechnology.

### Optimization for novel electron donors and acceptors

The range of elements, their oxidation states and the forms of organic carbon can be very different in type and abundance in extraterrestrial environments compared with environments on the Earth. For example, iron in iron‐rich meteorites is accessible to iron‐oxidizing microorganisms (e.g. Gronstal *et al*., 2009), but can be bound up in meteoritic iron‐nickel phases. On the Moon, what organics there are may be at low concentration in the form of ethylene, methanol, and methane (Colaprete *et al*., 2010). Carbonaceous asteroids contain a variety of organics including many tens of amino acids, many of them non‐proteinaceous species, and kerogen‐like insoluble carbon (Sephton, 2002). Although these organics are unusual, aerobic microorganisms have been shown to be able to grow on this carbonaceous chondritic material (Mautner, 1997).

Depending on the target body, we could imagine that one goal of biotechnology would be to incorporate secondary modifications into organisms to optimize their capacity to use distinctly extraterrestrial combinations of electron donors and acceptors or to access forms of carbon, for instance, that do not exist on Earth, such as certain meteoritic organics. This would require more research into both chemoautotrophic and heterotrophic pathways of energy acquisition using extraterrestrial materials, the identification of the molecular pathways involved and their incorporation into industrially useful organisms.

## Unusual microbial gaps – microbial biotechnology on Titan

Despite the generic outlines of the microbial biotechnological needs I have discussed, there are some exceptions to the ‘typical’ conditions to be found beyond Earth. A prominent example is the Saturnian moon Titan. It is the largest moon in the Solar System (5150 km diameter) and has a thick atmosphere primarily composed of nitrogen, methane, and hydrogen. Its brown colouration is accounted for by a high altitude hydrocarbon haze produced by photochemical reactions. These photochemically produced materials are usually called ‘tholins’. Its surface is covered in landforms and dunes made from these complex hydrocarbons. The surface of the Moon is carved by rivers of liquid methane/ethane made possible by its low surface temperature (−179.2°C). Its surface pressure is about 0.15 MPa, constituting a rare surface environment in the Solar System with a higher pressure than the surface of Earth.

The surface pressure on Titan is well below the highest pressure environments known to support microbial life on Earth (Bartlett, 2002) and thus pressure does not, on its own, limit life. However, the surface temperature of the moon is below the lower growth limit of known life. Although no known microbial life could grow on Titan’s surface, the microbial biotechnological potential of surface and atmospheric materials seems considerable. The substantial quantities of hydrogen in the atmosphere might be employed as a microbial electron donor in biotechnological processes and the liquid methane and ethane present on the surface could be collected and used in microbial oxidation with oxygen, the latter provided by water electrolysis.

**Fig. 3:**
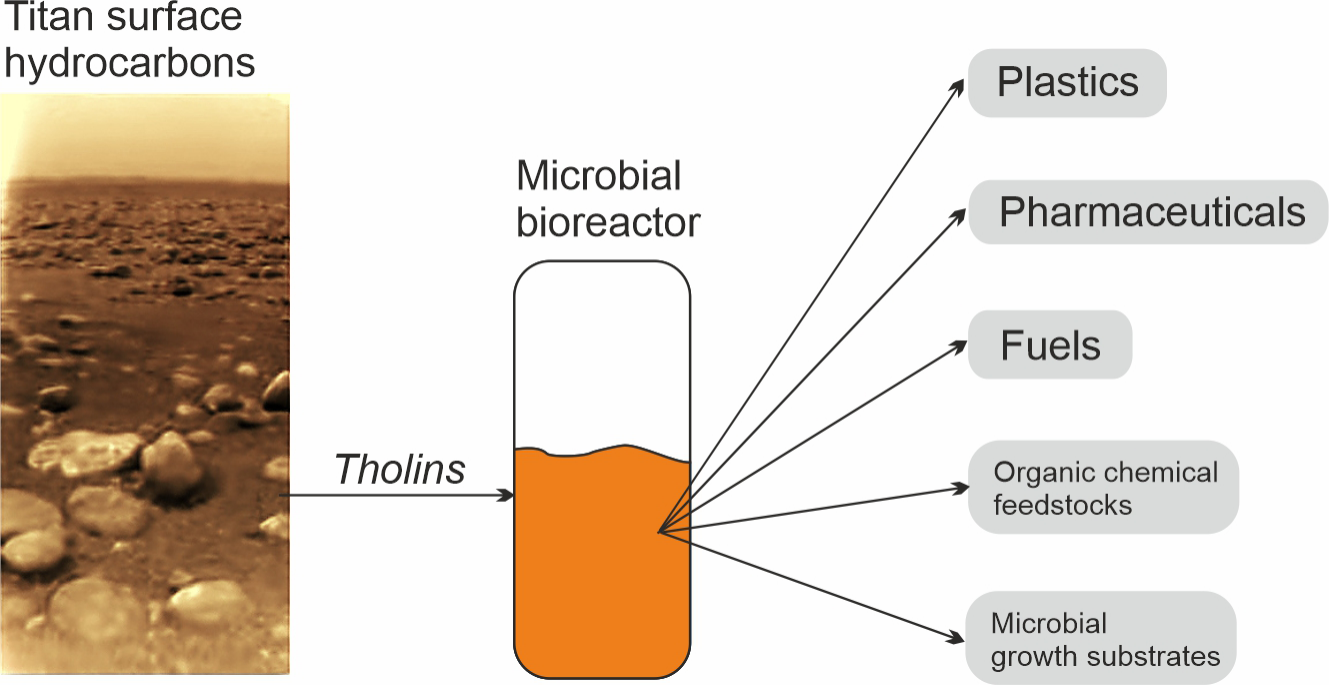
Tholin Microbial Biotechnology on Titan. Closing the gap between known microbial carbon metabolisms and carbon reservoirs beyond Earth. The vast hydrocarbon reservoirs on Titan (tholins) are microbially accessible forms of carbon. On the left is an image taken by the Huygens Lander (European Space Agency) in 2005 on the surface showing ‘sands’ of hydrocarbon material in among boulders of water ice. Organisms might be engineered to be capable of transforming these compounds in a heated bioreactor into useful end products, for example by fermentation and subsequence metabolic restructuring. Examples of desired end products are shown. ‘Feedstocks’ refers to generic molecular precursors (e.g. alcohols) used to make further products. These materials would supply a wide range of needs for carbon‐containing compounds in human settlements of the Outer Solar System, potentially making Titan a microbial foundry of human long‐term settlement in the far reaches of space.

In particular, the substantial hydrocarbon reservoirs (Clarke *et al*., 2010), which may be many hundreds of times greater in mass than all the known oil and gas reservoirs on Earth, raise intriguing possibilities for its potential biotransformation or fermentation into useful end products (Fig. 3). These complex tholins have been synthesized in the laboratory and have been shown to be metabolized by microorganisms (Stoker *et al*., 1990), suggesting that the material represents a potential vast reservoir of transformable microbial feedstock to supply the needs for carbon‐containing products in the outer Solar System. ‘Tholin microbial biotechnology’ might focus on the engineering of microorganisms to efficiently use tholins to produce high value products including plastics, pharmaceuticals, and chemical feedstocks to supply human settlement in the outer Solar System, thus closing the gap between existing microbial carbon metabolisms and optimization towards the carbon substrates available on Titan. We could imagine that the development of microbial strains and consortia capable of accessing such potentially recalcitrant organic carbon molecules would also aid in developing organisms capable of accessing the complex kerogen‐like organic carbon available in carbon molecule‐rich asteroids, such as carbonaceous chondrites.

This biotechnology programme would require growing microorganisms on tholins and identifying the metabolic pathways that efficiently degrade these molecules, engineering these pathways into new organisms that can be handled in industrial bioreactors and augmenting them with pathways that transform the tholin breakdown products into useful products. Organisms already known to degrade recalcitrant complex hydrocarbon molecules such as polycyclic aromatic hydrocarbons (Husain, 2008) could yield useful metabolic clues about how to optimize degradation of complex extraterrestrial hydrocarbons.

## Munchers in the service of space settlement–asteroid‐eating microbial biotechnology automatons

I want to close this perspective piece with an illustration of how we might bring these ideas together into a single machine that would be made possible by advances in space microbial biotechnology.

Consider the carbonaceous chondrites, asteroids that contain material that makes up ~ 5% of meteorite falls to Earth. These asteroids, which are some of the most primitive material in the Solar System, contain a wealth of potentially useful materials locked up in silicate minerals and rocks. They contain within them organic materials including amino acids, carboxylic acids, complex kerogen‐like compounds and many other compounds which could be extracted or transformed into useful products. Some (CI chondrites), contain high abundances of water (~ 20%) and others (CH chondrites) contain high abundances of metals (~ 40%). These asteroids are one type of material it would be useful to process into a wide range of products of use to human space settlement.

**Fig. 4:**
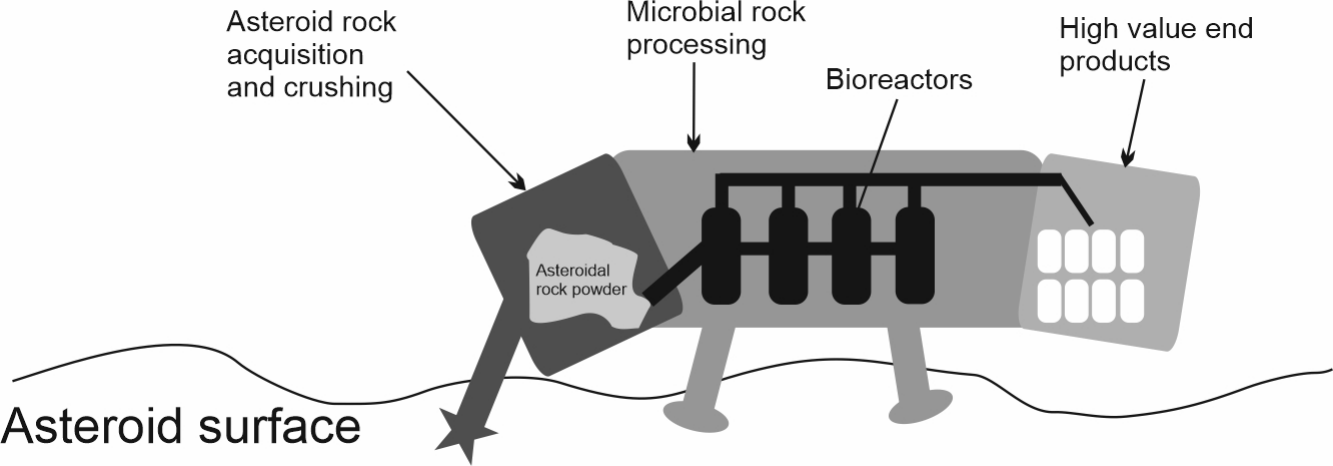
Munchers. Machines incorporating microbial reactors containing engineered microorganisms whose purpose is to collect and process raw asteroidal material into useful products for human space settlement. The general principle is depicted.

Imagine a robotic asteroid‐consuming machine which could be landed on these objects and would perform the microbial automated transformation of them into useful products. Let us call this machine a MUNCHer (Microbial UtilizatioN of Carbonaceous asteroids for High value products) (Fig. 4). The machine might vary from the size of a badger to a large truck, depending on the target object and production required. Many of the microbial biotechnological modifications I have discussed in this paper could be brought together into this machine which would contain a series of anaerobic microbial reactors. We might imagine a mandible‐like contraption on the front end of the muncher that breaks off the raw asteroidal material and crushes it, feeding it into the internal bioreactors. The muncher would be powered by nuclear and/or solar energy, which would power the machine’s movement as well as being used to crush the raw material and melt the asteroidal water to provide a fluid for the bioreactors. The reactors would contain strains or consortia of microorganisms with desiccation, freezing and high temperature tolerances, allowing for periodic disruption to their operation without detriment to their survival. Tolerances to high ion concentrations, potentially expected in asteroidal leachates, radiation resistance and the capacity to grow in low gravity would allow them to operate in this harsh environment.

The bioreactor series could perform a number of processes sequentially including: (i) metabolism and processing of asteroidal carbon compounds into useful high value organic products such as chemical feedstocks (for further chemical industrial processes), plastics and other manufacturing materials; (ii) the digestion of remaining rock and its biomining for metals and other essential elements; (iii) microbial transformation (such as redox transformation) of some of the extracted elements into useful industrial metals and compounds and (iv) separation and sequestering of rock from water and microbial bioremediation to produce clean potable water. These various materials would be collected at the end of the machine in a unit that could be detached and replaced with an empty unit, either robotically or by human‐tended operations.

The bioreactors within the muncher can be considered as a type of ‘gut’ whose purpose is to use microbial biotechnology to transform crushed raw asteroidal material fed in at one end into useful products secreted from the other end. The bioreactors are designed to be ‘plug and play’ whereby different microbial strains or consortia can be added depending on the products required and the exact composition of the asteroid to be disassembled. All of the microorganisms used contain the ‘primary’ modifications identified in Fig. 2, with ‘secondary’ modifications added, which would be determined by the expected physical and chemical environment on a given asteroid target. Munchers would be mass produced, allowing for microbial processing of large quantities of material across the Solar System. They might also be deployed to the surfaces of bodies other than asteroids, such as planets, moons and Kuiper Belt objects.

The Munchers are not a realistic proposition for the next 15 years, but ground work in developing microorganisms that they could eventually contain can be achieved. The munchers illustrate the potential long‐term vision that might motivate space microbial biotechnology within the near‐term.

## Conclusions

‘Mind the gap’ is an invocation to London tube travellers not to fall into the gap between the platform and the train. As humans move beyond Earth, so too microbiologists who plan to use microorganisms to carry out useful industrial processes must beware of the gap between natural evolutionarily derived microbial capabilities and the physical and chemical extremes to be encountered. In this perspectives piece, I have suggested that despite the varied pantheon of places that we might eventually settle in space, there are core challenges that are similar almost everywhere. At the moment, we do not know how universal these genetic modifications can be across taxa and how much must be fine‐tuned to be taxon‐specific. These ‘primary’ genetic modifications required for most space locations, such as the need for radiation resistance, could be augmented by ‘secondary’ genetic modifications that focus on specific capabilities needed in particular environments, such as UV radiation resistance in microbes used in photosynthetic applications. Closing the microbial gap constitutes a key challenge in space microbial biotechnology in the coming decades. A programme of microbial biotechnology should seek to systematically identify the genes required to cope with the key extremes to be found beyond the Earth, determine the minimum requisite changes to realize these resistant capacities, and develop the genetic engineering or synthetic biological methods and chassis organisms into which these capabilities can be incorporated, together with the genes for the industrial process of interest. As this perspectives piece illustrates, there is great deal that we do not know and much work to do to elucidate the mechanisms of interest. With the products of such a programme in hand, humanity will be equipped with the microbial biotechnological capacity to make full use of the resources of the Solar System to support a long‐term self‐sustaining human presence beyond Earth.

## Funding Information

This work was conducted under Science and Technology Facilities (STFC) grant no: ST/V000586/1.

## Conflict of interest

None declared.
